# Acute kidney injury in coronavirus disease: a comparative study of the two waves in Brazil

**DOI:** 10.31744/einstein_journal/2024AO0687

**Published:** 2024-09-25

**Authors:** Luis Eduardo Magalhães, Ana Júlia Favarin, Pedro Andriolo Cardoso, Bruna Kaori Yuasa, Welder Zamoner, André Luís Balbi, Daniela Ponce

**Affiliations:** 1 Universidade Estadual Paulista Faculdade de Medicina de Botucatu Botucatu SP Brazil Faculdade de Medicina de Botucatu, Universidade Estadual Paulista, Botucatu, SP, Brazil.

**Keywords:** Coronavirus infections, COVID-19, Prognosis, Acute kidney injury, Receptor cross-talk, Incidence, Mortality, Brazil

## Abstract

**Objective:**

This study aimed to evaluate the incidence of acute kidney injury in hospitalized Brazilian patients with COVID-19 and identify the risk factors associated with its development and prognosis during the two waves of the disease.

**Methods:**

We performed a prospective cohort study of hospitalized patients with COVID-19 at a public university hospital in São Paulo from March 2020 to May 2021.

**Results:**

Of 887 patients hospitalized with COVID-19, 54.6% were admitted to the intensive care unit. The incidence of acute kidney injury was 48.1%, and the overall mortality rate was 38.9%. Acute kidney replacement therapy was indicated for 58.8% of the patients. The factors associated with acute kidney injury were diuretic use (odds ratio [OR] 2.2, 95%CI= 1.2-4.1, p=0.01), mechanical ventilation (OR= 12.9, 95%CI= 4.3-38.2, p<0.0001), hematuria(OR= 2.02, 95%CI= 1.1-3.5, p<0.0001), chronic kidney disease (OR= 2.6, 95%CI= 1.2-5.5, p=0.009), age (OR= 1.03, 95%CI= 1.01-1.07, p=0.02), and elevated creatine phosphokinase (OR= 1.02, 95%CI= 1.01-1.07, p=0.02) and D-dimer levels (OR= 1.01, 95%CI= 1.01-1.09, p<0.0001). Mortality was higher among those with acute kidney injury (OR= 1.12, 95%CI= 1.02-2.05, p=0.01), elevated Sequential Organ Failure Assessment Scores (OR= 1.35, 95%CI= 1.1-1.6, p=0.007), elevated Acute Tubular Necrosis-Injury Severity Score (ATN-ISS; (OR= 96.4, 95%CI= 4.8-203.1, p<0.0001), and who received mechanical ventilation (OR= 12.9, 95%CI= 4.3-38.2, p<0.0001). During the second wave, the number of cases requiring mechanical ventilation (OR= 1.57, 95%CI= 1.01-2.3, p=0.026), with proteinuria (OR= 1.44, 95%CI= 1.01-2.1, p=0.04), and with higher ATN-ISS Scores (OR= 40.9, 95%CI= 1.7-48.1, p=0.04) was higher than that during the first wave.

**Conclusion:**

Acute kidney injury was frequent in hospitalized patients with COVID-19, and the second wave was associated with greater severity. However, mortality rates were similar between the two periods, which may reflect both the effectiveness of vaccines and the constant learning that frontline professionals gained throughout the pandemic to provide greater support to their patients.

**Registry of Clinical Trials:**

RBR-62y3h7

## In Brief

Magalhães et al. demonstrated that the incidence of acute kidney injury was high in hospitalized patients with COVID-19 and that the second wave was associated with greater severity; however, the mortality rates were similar between the two periods. This may reflect both the effectiveness of vaccines and the constant learning that frontline professionals gained throughout the pandemic to provide greater support to their patients.

## Highlights

◼ Renal involvement was frequent in patients with COVID-19 and related to worse outcomes.
◼ Diuretic use, mechanical ventilation, proteinuria, hematuria, age, and creatine phosphokinase and D-dimer levels were risk factors for acute kidney injury.
◼ Acute kidney injury, mechanical ventilation, elevated SOFA Score, and elevated ATN-ISS were associated with mortality.
◼ The second wave was associated with greater severity; however, the mortality rates were similar between the two periods.
◼ This may reflect the effectiveness of vaccines and the constant learning that frontline professionals gained throughout the pandemic.

## Graphical abstract

**Figure:**
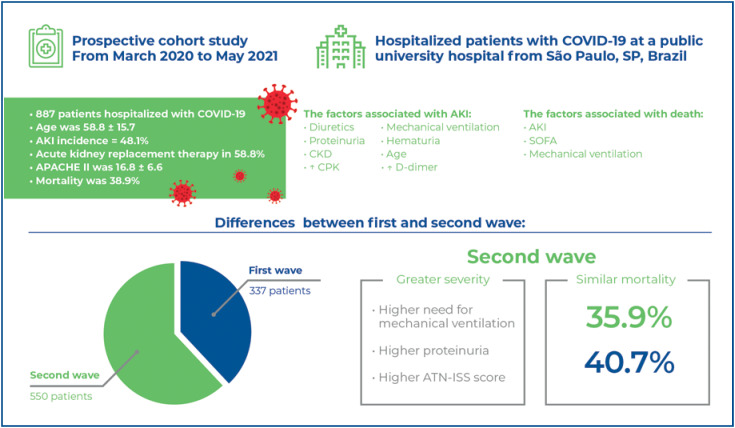

## INTRODUCTION

Coronavirus disease 2019 (COVID-19), caused by severe acute respiratory syndrome coronavirus 2 (SARS-CoV-2), was declared a pandemic by the World Health Organization in March 2020.

The clinical spectrum of COVID-19 ranges from typical and atypical symptoms of upper respiratory tract infection to more severe complications such as pneumonia and acute respiratory distress syndrome, which usually require intensive care.^(1,2)^

Other complications include circulatory shock, heart failure, and acute kidney injury (AKI). Using a pathophysiological model of cross-talk, few authors hypothesized that severe COVID-19 is associated with immune dysregulation, cytokine storm, and systemic inflammation.^(2,3)^ Renal involvement is frequent (4-37%), especially among critically ill patients, and is a factor related to worse outcomes.^(3-6)^ This incidence is associated with age, disease severity, and ethnicity.

Acute kidney injury is characterized by a rapid decline in renal function, with consequent accumulation of nitrogenous wastes and occurrence of hydroelectrolytic and acid-base disorders.^(3-8)^ Besides direct virus-induced tissue damage, the involvement of organs in COVID-19 may occur secondary to inflammation, endothelial dysfunction, and hypercoagulability.

Acute kidney injury has been associated with increased mortality among hospitalized patients with COVID-19. According to a recent meta-analysis, the incidence of AKI in patients with COVID-19 was 8.9%.^(9)^However, statistical heterogeneity was observed among the included studies. Other meta-analyses have showed that male sex and diabetes are associated with a higher AKI and mortality rate among patients with COVID-19.^(10,11)^ Studies from the USA and Europe have reported pooled incidences of 28.6% and 7.7%for AKI, respectively, and AKI has been identified as a predictor of fatality and severe COVID-19.^(12,13)^

The pathophysiology of AKI in patients with COVID-19 is multifactorial. The effects of a SARS-CoV-2 infection on the renal tissues can be direct or indirect.^(5,10)^ Direct effects include endothelial damage due to viral entry, local inflammation, and collapsing glomerulopathy, whereas indirect effects include sepsis, adverse effects due to nephrotoxic drug use, and systemic inflammation-also known as a cytokine storm.

Although there lies ample literature on the association between respiratory failure and AKI, few studies have elucidated the renal repercussions caused by the novel coronavirus in view of its recent discovery.

## OBJECTIVE

To evaluate the incidence of acute kidney injury in hospitalized Brazilian patients with COVID-19 during the two waves of the disease and to identify risk factors associated with its onset and prognosis between the two periods.

## METHODS

The research was performed following current regulations, and written informed consent was obtained from all participants or their legal guardians.

A prospective cohort study of hospitalized patients diagnosed with COVID-19, confirmed using real-time polymerase chain reaction for SARS-CoV-2, was performed in clinical wards and intensive care units (ICUs) of a public and tertiary university hospital in São Paulo, Brazil, from March to December 2020 (first wave) and January to May 2021 (second wave). Patients were hospitalized until the clinical outcome (discharge or mortality) was met, AKI diagnosis was assessed, and risk factors were identified through the collection of information from electronic medical records including those of their diagnosis, mortality, and indication for acute kidney replacement therapy.

Clinical and laboratory data were collected during hospitalization. Renal function was evaluated daily by measuring the serum creatinine levels and urine output. AKI was identified according to the Kidney Disease: Improving Global Outcomes (KDIGO) definition: an increase in serum creatinine level >0.3mg/dL within 48 hours or by 50% within 7 days; AKI was staged as one of the three KDIGO categories.^(6)^ For the detection of proteinuria or hematuria, a semi-quantitative dipstick test was performed; data were requested at admission for all patients and during hospitalization for patients without proteinuria at admission. The indications for acute kidney replacement therapy were uremia or azotemia (blood urea nitrogen >100mg/dL), fluid overload (after diuretic use), electrolyte imbalance (K >6.5mEq/L after clinical treatment), acid-base disturbance (pH <7.1 and bicarbonate <10mEq/L after clinical treatment), and metabolic and fluid demand-to-capacity imbalance.^(13,14)^ The demand is determined by the severity of the acute illness and solute and fluid burdens. The demand capacity balance is dynamic and varies with the course of critical illness. When renal capacity decreased and the patient failed to cope with the demands, acute kidney replacement therapy was initiated.

Using the study protocol, the data were entered into an electronic spreadsheet, eliminating any typographical errors. Analyses were performed using SPSS version 20 (IBM Corp., Armonk, NY) or SigmaStat 3.5 (Systat Software, San Jose, CA). The frequency or central tendency and dispersion measures were calculated for categorical and continuous variables, respectively, with AKI as the outcome variable. The χ^2^ test was used to compare categorical variables, and the *t*-test was used for continuous variables.

A multivariate analysis was performed through the construction of a logistic regression model with calculations of the odds ratio (OR), including all the independent variables that showed association with the outcome in the model, with p≤0.05. A similar procedure was performed by establishing the occurrence of mortality and indication for acute kidney replacement therapy as dependent variables.

This study was approved by the local Ethics Committee of *Faculdade de Medicina de Botucatu* (CAAE: 30451520.6.0000.5411; #4.003.880).

## RESULTS

**Figure 1:**
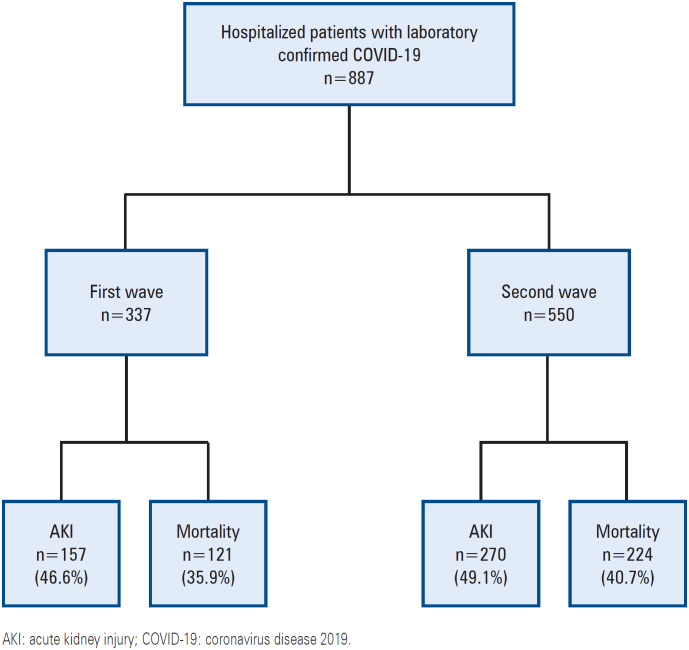
Classification of patients

Between 2020 and 2021, 887 patients with a confirmed diagnosis of COVID-19 were hospitalized at our hospital (Figure 1). The mean age was 58.8±15.7 years; 52.3% were men, 59.1% had hypertension, 54.6% were admitted to the ICU, and 45.4% were admitted to the ward. The overall incidence of AKI was 48.1%; AKI was more frequent among patients admitted to the ICU than among those admitted to the ward (83.8% *versus* 17.1%, p<0.0001). The mean time for AKI diagnosis was 6 days, and KDIGO stage 3 was most frequently diagnosed (60.2%). Acute kidney replacement therapy was indicated for 263 (58.8%) patients.

Factors associated with AKI development were advanced age (61.5±14.4 *versus* 56.3±16.3 years, p<0.0001), ICU admission (83.8% *versus* 27.6%, p<0.0001), mechanical ventilation (81.5% *versus* 11.5%, p<0.0001), vasoactive drug use (VAD; 80.1% *versus* 12.6%, p<0.0001), proteinuria (68.2% *versus* 33.9%, p<0.0001), and hematuria (75% *versus* 40.5%, p<0.0001) (Table 1).

**Table 1:** Clinical and laboratory characteristics of hospitalized patients with or without acute kidney injury

Variables	General (n=887)	Without AKI (n=460)	With AKI (n=427)	p value
First wave (%)	337 (38)	180 (39.1)	157 (36.8)	0.469
Second wave (%)	550 (62)	280 (60.8)	270 (63.2)	0.469
Male sex (%)	464 (52.3)	229 (49.8)	235 (55)	0.118
Caucasian ethnicity (%)	750 (84.6)	403 (87.6)	347 (82.2)	0.025
Age (years)*	58.8±15.7	56.3±16.3	61.5±14.4	<0.0001
Arterial hypertension (%)	524 (59.1)	235 (51.1)	289 (67.7)	<0.0001
ACE inhibitor use (%)	377 (42.5)	172 (37.4)	205 (48)	0.001
Diuretic use (%)	214 (24.1)	91 (19.8)	123 (28.8)	0.002
CVD (%)	157 (17.7)	76 (16.5)	81 (19)	0.34
Diabetes (%)	229 (33.7)	131 (28.5)	168 (39.3)	0.001
Obesity (%)	288 (32.5)	118 (25.7)	170 (39.8)	<0.0001
Dyslipidemia (%)	190 (21.4)	77 (16.7)	113 (26.5)	<0.0001
Lung disease (%)	87 (9.8)	44 (9.6)	43 (10.1)	0.8
Smoking (%)	232 (26.2)	125 (27.2)	107 (25.1)	0.48
CKD (%)	118 (13.3)	48 (10.4)	70 (16.4)	0.009
GFR (mL/min/1.73 m^2^)	90.8±29.9	98.9±26.7	82.7±30.5	<0.0001
Dialysis (%)	263 (29.7)	12 (2.6)	251 (58.8)	<0.0001
ATN-ISS*	0.7±0.2	0.8	0.7±0.2	0.491
CPK**	117 (52.5-436)	68 (37-146)	274 (84-841)	<0.0001
D-dimer**	2509 (1133.7-10956.2)	1485 (859-3144)	7533 (2137-15527)	<0.0001
Mechanical ventilation (%)	401 (45.2)	53 (11.5)	348 (81.5)	<0.0001
Vasoactive drug use (%)	400 (45.1)	58 (12.6)	342 (80.1)	<0.0001
ICU admission (%)	484 (54.6)	126 (27.6)	358 (83.8)	<0.0001
APACHE*	16.8±6.6	12.6±5.3	18.3±6.3	<0.0001
SOFA*	7±3.8	4.8±3.1	7.7±3.7	<0.0001
Hematuria (%)	508 (57.2)	186 (40.5)	322 (75)	p<0.0001
Proteinuria (%)	447 (50.4)	155 (33.9)	292 (68.2)	p<0.0001
Mortality (%)	345 (38.9)	52 (11.3)	293 (68.6)	<0.0001

* Mean±SD

** Median (interquartile range).

AKI: acute kidney injury; ACE: angiotensin-converting enzyme; CKD: chronic kidney disease; CVD: cardiovascular disease; GFR: glomerular filtration rate; CPK: creatine phosphokinase; ICU: intensive care unit; ATN-ISS: Acute Tubular Necrosis-Injury Severity Score; APACHE II: Acute Physiology and Chronic Health Evaluation; SOFA: Sequential Organ Failure Assessment.

Diuretic use (OR= 2.2, 95% confidence interval [CI]= 1.2-4.1, p=0.01), mechanical ventilation (OR= 12.9, 95%CI= 4.3-38.2, p<0.0001), hematuria (OR= 2.02, 95%CI= 1.1-3.5, p=0.01), chronic kidney disease (CKD; OR= 2.6, 95%CI= 1.2-5.5, p=0.009), older age (OR= 1.03, 95%CI= 1.01-1.07], p=0.02), and elevated creatine phosphokinase (CPK; OR= 1.02, 95%CI= 101-107, p=0.02) and D-dimer (OR= 1.01, 95%CI= 101-1.09, p<0.0001) levels were risk factors for AKI. Table 2 shows the factors associated with AKI in the multivariate analysis.

**Table 2:** Logistic regression of variables associated with acute kidney injury

Variables	Odds ratio	95% confidence interval	p value
Diuretic use	2.2	1.2-4.1	0.01
Dyslipidemia	0.54	0.3-1.05	0.07
Vasoactive drug use	2.7	0.9-7.7	0.06
Mechanical ventilation	12.9	4.3-38.2	<0.0001
Chronic kidney disease	2.6	1.2-5.5	0.009
Hematuria	2.02	1.1-3.5	0.01
Age	1.03	1.01-1.07	0.02
Creatine phosphokinase	1.02	1.01-1.07	0.02
D-dimer	1.01	1.01-1.09	<0.0001

The overall mortality rate was 38.9% (345 patients), which was higher among ICU patients (63.2 *versus* 9.6%, p<0.0001). Factors associated with mortality were advanced age (64.11±14.2 *versus* 55.5±15.7 years, p<0.0001), ICU admission (88.7% *versus* 32.8%, p<0.0001), VAD use (85.5% *versus* 19.4%, p<0.0001), mechanical ventilation (86.4% *versus* 19%, p<0.0001), kidney acute support (61.4% *versus* 9.4%, p<0.0001), AKI (84.9% *versus* 24.7%, p<0.0001), KDIGO stage 3 (59.7% *versus* 9.4%, p<0.0001), proteinuria (70.9% *versus* 38.1%, p<0.0001), and hematuria (75.3% *versus* 46.9%, p<0.0001) (Table 3).

**Table 3:** Clinical and laboratory characteristics of hospitalized patients with respect to mortality

Variables	No mortality (n=542)	Mortality (n=345)	p value
First wave (%)	216 (39.9)	121 (35.1)	0.153
Second wave (%)	326 (60.1)	224 (64.9)	0.153
Male sex (%)	270 (49.8)	194 (56.2)	0.062
Caucasian ethnicity (%)	464 (85.9)	286 (83.6)	0.351
Age (years)*	55.5±15.7	64.11±14.2	<0.0001
Arterial hypertension (%)	284 (52.4)	240 (69.5)	<0.0001
ACE inhibitor use (%)	210 (38.7)	167 (48.4)	0.005
Diuretic use (%)	122 (22.5)	92 (26.7)	0.158
CVD (%)	82 (15.1)	75 (21.7)	0.012
Diabetes (%)	164 (30.3)	135 (39.1)	0.006
Obesity (%)	155 (29.2)	130 (37.7)	0.023
Dyslipidemia (%)	106 (19.6)	84 (24.3)	0.09
Lung disease (%)	52 (9.6)	35 (10.1)	0.788
Smoking (%)	138 (25.5)	94 (27.2)	0.617
CKD (%)	65 (12)	53 (15.4)	0.15
AKI (%)	134 (24.7)	293 (84.9)	<0.0001
KDIGO stage 3 (%)	51 (9.4)	206 (59.7)	<0.0001
GFR (mL/min/1.73 m^2^)	97.2±27.6	81.5±30.4	<0.0001
Dialysis (%)	51 (9.4)	212 (61.4)	<0.0001
ATN-ISS*	0.54±0.28	0.78±0.14	<0.0001
CPK**	78 (40-193)	293 (86-754)	<0.0001
D-dimer**	1683 (969-5707)	7433 (2116-14931)	<0.0001
Mechanical ventilation (%)	103 (19)	298 (86.4)	<0.0001
Vasoactive drug use (%)	105 (19.4)	295 (85.5)	<0.0001
ICU admission (%)	178 (32.8)	306 (88.7)	<0.0001
APACHE*	12.6±5.7	19.3±5.8	<0.0001
SOFA*	4.7±3.2	83±3.4	<0.0001
Hematuria	252 (46.9)	261 (75.3)	<0.0001
Proteinuria	209 (38.1)	245 (70.9)	<0.0001

* Mean±SD;

** Median (interquartile range).

AKI: acute kidney injury; ACE: angiotensin-converting enzyme; CKD: chronic kidney disease; CVD: cardiovascular disease; GFR: glomerular filtration rate; CPK: creatine phosphokinase; KDIGO: Kidney Disease: Improving Global Outcomes; ICU: intensive care unit; ATN-ISS: Acute Tubular Necrosis-Injury Severity Score; APACHE II: Acute Physiology and Chronic Health Evaluation; SOFA: Sequential Organ Failure Assessment.

Acute kidney injury (OR= 1.12, 95%CI= 1.02-2.05, p=0.01), mechanical ventilation (OR= 12.9, 95%CI= 4.3-38.2, p<0.0001), elevated SOFA Scores (OR= 1.35, 95%CI= 1.1-1.6, p=0.007), and elevated ATN-ISS (OR= 96.4, 95%CI= 4.8-203.1, p<0.0001) were associated with mortality (Table 4).

**Table 4:** Logistic regression of variables associated with death

Variables	OR	95%CI	p value
AKI	1.12	1.02-2.05	0.01
SOFA	1.35	1.1-1.6	0.007
ATN-ISS	96.4	4.8-203.1	<0.0001
Mechanical ventilation	12.9	4.3-38.2	<0.0001
Vasoactive drugs	1.06	0.96-5.5	0.09
Obesity	1.01	0.91-3.5	0.15
Age	1.02	0.99-1.54	0.11

Failure Assessment Score.

AKI: Acute kidney injury; ATN-ISS: Acute Tubular Necrosis-Injury Severity Score; SOFA: Sequential Organ.

Univariate analysis identified clinical and laboratory similarities and differences between patients hospitalized during the first and second waves of the pandemic (Table 5). During the first wave, the proportion of men (58.2% *versus* 48.7%, p=0.006) and D-dimer levels (1558-11221 *versus* 948-9938, p=0.009) were higher than those during the second wave. During the second wave, proteinuria (57% *versus* 45.2%, p=0.009), hematuria (63% *versus* 53.5%, p=0.033), increased use of dialysis (32.4% *versus* 25.2%, p=0.024), mechanical ventilation (48.4% *versus* 40.1%, p=0.016), VAD use (48% *versus* 40.4%, p=0.026), and higher ATN-ISS (0.76±0.2 *versus* 0.63±0.24, p<0.001) were increased than those during the first wave. During the first wave, no participants were vaccinated; in contrast 14% were vaccinated during the second wave (one dose).

**Table 5:** Clinical and laboratory characteristics of hospitalized patients during the first and second waves

Variables	First wave (n=337)	Second wave (n=550)	p value
Male sex (%)	196 (58.2)	268 (48.7)	0.006
Caucasian ethnicity (%)	271 (80.4)	479 (87.9)	0.002
Age (years)*	59.5±16.2	58.4±15.3	0.32
Vaccinated patients (%)	0 (0)	77 (14)	<0.001
Arterial hypertension (%)	203 (60.2)	321 (58.4)	0.58
ACE inhibitor use (%)	145 (43)	232 (42.2)	0.8
Diuretic use (%)	93 (27.6)	121 (22)	0.059
CVD (%)	60 (17.8)	97 (17.6)	0.95
Diabetes (%)	119 (35.3)	180 (32.7)	0.43
Obesity (%)	98 (29.1)	190 (34.5)	0.17
Dyslipidemia (%)	75 (22.3)	115 (20.9)	0.63
Lung disease (%)	29 (8.6)	58 (10.5)	0,34
Smoking (%)	79 (23.4)	153 (27.8)	0.25
CKD (%)	44 (13.1)	74 (13.5)	0.86
AKI (%)	157 (46.6)	270 (49.1)	0.47
KDIGO stage 3 (%)	84 (24.9)	173 (31.5)	0.13
GFR (mL/min/1.73 m^2^)	89±29.3	91.8±29.9	0.25
Dialysis (%)	85 (25.2)	178 (32.4)	0.024
ATN-ISS*	0.63±0.24	0.76±0.2	<0.0001
CPK**	99 (47-407)	146 (62-495)	0.083
D-dimer**	4268 (1558-11221)	1940 (948-9938)	0.009
Mechanical ventilation (%)	135 (40.1)	266 (48.4)	0.016
Vasoactive drug use (%)	136 (40.4)	264 (48)	0.026
ICU admission (%)	175 (51.9)	309 (56.2)	0.21
APACHE II*	17.8±7.5	16.2±5.9	0.02
SOFA*	7.7±4.1	6.6±3.5	0.003
Proteinuria	151 (45.2)	313 (57)	0.009
Hematuria	179 (53.5)	346 (63)	0.033
Mortality (%)	121 (35.9)	224 (40.7)	0.15

* Mean±SD

** Median (interquartile range).

AKI: acute kidney injury; ACE: angiotensin-converting enzyme; CKD: chronic kidney disease; CVD: cardiovascular disease; GFR: glomerular filtration rate; CPK: creatine phosphokinase; ICU: intensive care unit; KDIGO: Kidney Disease: Improving Global Outcomes; ATN-ISS: Acute Tubular Necrosis-Injury Severity Score; APACHE II: Acute Physiology and Chronic Health Evaluation; SOFA: Sequential Organ Failure Assessment.

Table 6 shows variables with differences during the second wave of the pandemic. Male sex (OR= 0.51, 95%CI= 0.35-0.74, p<0.0001) and Caucasian ethnicity (OR= 0.47, 95%CI= 0.2-0.8, p=0.006) were less prevalent during the second wave, while mechanical ventilation (OR= 1.57, 95%CI= 101-2.3, p=0.026), proteinuria (OR= 1.44, 95%CI= 101-2.1, p=0.04), D-dimer level (OR= 1.09, 95%CI= 1.02-1.1, p=0.02), and elevated ATN-ISS (OR= 40.9, 95%CI= 1.7-48.1, p=0.04) were more prevalent during the second wave.

**Table 6:** Logistic regression analysis of patient variables during the second wave

Variables	Odds ratio	95%CI	p value
Male sex	0.51	0.35-0.74	<0.0001
Caucasian race	0.47	0.2-0.8	0.006
Mechanical ventilation	1.57	1.01-2.3	0.026
Proteinuria	1.44	1.01-2.1	0.04
D-dimer	1.09	1.02-1.1	0.02
Acute Tubular Necrosis-Injury Severity Score	40.9	1.7-48.1	0.04

95%CI: 95% confidence interval.

## DISCUSSION

This study describes the first and second waves of the COVID-19 pandemic at a public university hospital in São Paulo, Brazil, which serves as a reference for 28 municipalities in the region with more than 2 million inhabitants.^(14)^ During this period, 887 patients diagnosed with COVID-19 were hospitalized; 54.6% were admitted to the ICU and 45.4% to the ward. The overall incidence of AKI was 48.1%, with a mean diagnosis time of 6 days. This incidence was higher than that reported in the literature.

Chinese studies^(15-21)^ have reported a low and variable incidence of AKI in hospitalized patients (0.5-7%) and a higher incidence in severe COVID19 cases (2.9-19%). European and North American studies^(22-27)^ have reported an AKI incidence of 20-40% in patients hospitalized with COVID-19. In all cohorts, AKI occurred between days 7 and 14 of illness; it was associated with higher hospital mortality and was decisive in the prognosis of these patients.^(25)^ Brazil is a large country with several vulnerable groups, in addition to an emerging economy and fragile social protection, which may have contributed to the increased demand for health services and the development of serious forms of COVID-19.^(28)^

SARS-CoV-2 comprises a spike protein that binds to the angiotensin-converting enzyme 2 (ACE2) receptor present in host cells, enabling its activation and cleavage by transmembrane proteases, culminating in the release of fusion peptides by the virus. ACE2 is highly expressed in the mouth, tongue, and alveolar epithelial cells. In the kidneys, it is highly expressed in proximal tubule cells and, to a lesser extent, in podocytes.^(16,25,26)^ Thus, the higher AKI incidence in European and American countries could be associated with the higher expression of ACE2 in podocytes and proximal tubules in Western individuals than in Eastern individuals, as identified in normal kidneys and described by Pan et al.^(26)^ However, other studies did not identify SARS-CoV-2 in renal biopsy/autopsy tissue samples.^(27)^

Acute kidney injury is a complex disorder characterized by the degradation of renal function over hours to days, resulting in a temporary decrease or interruption of renal capacity to promote the excretion of nitrogenous products and hydroelectrolytic homeostasis of the body, resulting in volume overload.^(29)^ Its incidence in hospitalized patients varies between 5 and 7%; according to other studies, it is higher in ICU patients (approximately 50%). Despite technological advances and the reduction in mortality rate in the last decade, AKI prognosis remains severe, and the mortality rate remains high, especially in patients requiring dialysis (up to 62%).^(30-38)^

Logistic regression analysis revealed that the factors associated with the development of AKI in patients hospitalized with COVID-19 were diuretic use, dyslipidemia, mechanical ventilation, VAD use, CKD, proteinuria, hematuria, older age, and elevated CPK and D-dimer levels.

In a Brazilian study, Bucuvic et al.^(35)^ reported that 62% of patients diagnosed with AKI were of male sex, 65.2% were aged >60 years, 61.9% had *diabetes mellitus*, 44.4% had hypertension, and 21.9% had CKD. Ponce et al.^(36)^ performed a large retrospective observational study investigating the epidemiology of AKI and its effect on patient outcomes over time. For comparison purposes, patients were divided into two groups according to the year of follow-up: 2011-2014 and 2015-2018. The authors evaluated 5,428 patients with AKI. Three (50.6%) patients had stage 3 AKI, and the mortality rate was 34.3% (1,865 patients). Dialysis was indicated for 928 patients (17.1%). Patient survival improved during the study periods, and patients treated during 2015-2018 had a relative risk mortality reduction of 0.89 (95%CI= 0.81-0.98, p=0.02). The independent risk factors for mortality were sepsis, age >65 years, ICU admission, KDIGO stage 3, recurrent AKI, no metabolic and fluid demand-to-capacity imbalance (as a dialysis indication), and treatment period.

A recent systematic review showed that 40%, 61.4%, 57.1%, and 22.2% of patients with COVID-19 and AKI had a history of *diabetes mellitus*, hypertension, hyperlipidemia, and CKD, respectively.^(37)^ Based on a growing consensus and evidence, factors such as older age, diabetes, hypertension, cardiovascular disease, high body mass index, CKD, immunosuppression for any reason, and smoking are potential risk factors for COVID-19-associated AKI.^(38-40)^ Some laboratory parameters, including leukocytosis; lymphopenia; elevated levels of C-reactive protein and ferritin; hematuria; and proteinuria,^(41-44)^ were also associated with COVID-19-associated AKI; their incidence was 100%, 72.2%, 92.7%, 88.9%, 61.8%, and 97.4%, respectively.

Acute kidney injury is associated with worse clinical outcomes. A 2015 international multicenter study^(37)^ involving 1,032 ICU patients showed that AKI was independently associated with higher mortality at all stages, with the following ORs: 1.7 for KDIGO stage 1 and 6.9 for KDIGO stage 3. In ICU patients, AKI is associated with a longer duration of mechanical ventilation, VAD use, and an increased length of hospital stay, with acute kidney replacement therapy required in 50% of cases.

The data presented in our study revealed an overall mortality rate of 38.9%, which was higher among ICU patients (63.2% *versus* 9.6%, p<0.0001). Factors associated with mortality were AKI, KDIGO stage 3, arterial hypertension, VAD use, mechanical ventilation, proteinuria, high D-dimer level, elevated SOFA Scores, and elevated ATN-ISS.

In terms of prognosis, previous studies have shown that ICU hospitalization and the need for assisted ventilation are commonly reported in 86.7% and 87.5% of AKI patients, respectively. In patients with COVID-19 and AKI, the overall hospital mortality rate was approximately 84%, similar to that in early reports from developing countries.^(45,46)^ AKI is an independent risk factor for increased mortality in critically ill patients with diseases, including COVID-19.^(47)^ Kidney involvement has also been reported as an indicator of poor prognosis, regardless of the initial COVID-19 severity,^(48)^ which suggests that early detection and treatment of renal abnormalities improve the vital prognosis of patients with COVID-19.

Many factors contribute to the high mortality rate of AKI in patients with COVID-19, especially the lack of identification of risk factors for the development of this pathology at the time as well as the lack of knowledge about factors associated with mortality.^(49-55)^

However, mechanical ventilation, mainly associated with renal and/or pulmonary involvement, may predispose patients to hospital infections that contribute to higher mortality. When intubated patients are receiving mechanical ventilation, lung defense mechanisms are altered by the underlying disease or by the loss of protection of the upper airways, such as the loss of an intact cough reflex, which may result in pulmonary hypersecretion or an increase in the frequency of respiratory infections with high morbidity and mortality rates.

Notably, proteinuria, as highlighted in our results, was shown to be an important risk factor associated with the development of AKI, which is the main pathophysiological basis for the hypothesis of direct damage caused by SARS-CoV-2 in tubular epithelial cells and renal podocytes. Therefore, proteinuria in patients with COVID-19 may be associated with viral cytopathic effects, which reduce filtration and protein reabsorption, resulting in tubular injury, or even have a glomerular origin, especially in patients who develop acute glomerulopathy.^(5,8,26)^

Using logistic regression, this study identified similarities and clinical and laboratory differences between patients hospitalized during the first and second waves of the pandemic. During the first wave, a predominance of men and Caucasians was observed, while during the second wave, a predominance of cases requiring mechanical ventilation, with proteinuria, and with higher D-dimer levels and elevated ATN-ISS were observed.

Thus, it can be inferred that clinically more severely ill patients were hospitalized during the second wave, with a greater need for mechanical ventilation and greater AKI severity, despite the similar incidence of AKI and mortality between waves, which may reflect both the effectiveness of the SARS-CoV-2 vaccines and the constant learning that frontline professionals gained throughout the pandemic to provide greater support to their patients.

The limitation of this study was that data were collected from a single center using electronic medical records. However, this study represents important data regarding the epidemiological profile of AKI associated with COVID-19 in Brazil, a country with continental characteristics and a heterogeneous and vulnerable population.

## CONCLUSION

Acute kidney injury associated with severe COVID-19 was more frequent than that already reported in Chinese, European, and North American studies. The risk factors for acute kidney injury were diuretic use, mechanical ventilation, vasoactive drug use, dyslipidemia, proteinuria, hematuria, chronic kidney disease, older age, and elevated creatine phosphokinase and D-dimer levels. Mortality rate was high in this population and higher in patients with hypertension; mechanical ventilation; proteinuria; acute kidney injury (mainly KDIGO stage 3); and elevated D-dimer levels, SOFA Scores, and ATN-ISS. Mortality rates were similar between waves, which may reflect both the effectiveness of vaccines against SARS-CoV-2 and the constant learning that frontline professionals gained throughout the pandemic to provide greater support to their patients.

## TRANSPARENCY DECLARATION

The lead authors confirm that the manuscript is an honest, accurate, and transparent account of the study being reported; that no important aspects of the study have been omitted; and that any discrepancies from the study as originally planned (and, if relevant, registered) have been explained.

## DATA AVAILABILITY STATEMENT

Raw data supporting the conclusions of this study are available from the authors without undue reservation.

## References

[1] Lentini P, de Cal M, Clementi A, D’Angelo A, Ronco C (2012). Sepsis and AKI in ICU Patients: The Role of Plasma Biomarkers. Crit Care Res Pract.

[2] Singer M, Deutschman CS, Seymour CW, Shankar-Hari M, Annane D, Bauer M (2016). The Third International Consensus Definitions for Sepsis and Septic Shock (Sepsis-3). JAMA.

[3] Zarjou A, Agarwal A (2011). Sepsis and acute kidney injury. J Am Soc Nephrol.

[4] Ronco C, Reis T, Husain-Syed F (2020). Management of acute kidney injury in patients with COVID-19. Lancet Respir Med.

[5] Pecly IM, Azevedo RB, Muxfeldt ES, Botelho BG, Albuquerque GG, Diniz PH (2021). A review of Covid-19 and acute kidney injury: from pathophysiology to clinical results. J Bras Nefrol.

[6] Kidney Disease: Improving Global Outcomes (KDIGO) Acute Kidney Injury Work Group (2012). KDIGO Clinical Practice Guideline for Acute Kidney Injury. Kidney Int Suppl.

[7] Li R, Pei S, Chen B, Song Y, Zhang T, Yang W (2020). Substantial undocumented infection facilitates the rapid dissemination of novel coronavirus (SARS-CoV-2). Science.

[8] Ghinai I, McPherson TD, Hunter JC, Kirking HL, Christiansen D, Joshi K, Rubin R, Morales-Estrada S, Black SR, Pacilli M, Fricchione MJ, Chugh RK, Walblay KA, Ahmed NS, Stoecker WC, Hasan NF, Burdsall DP, Reese HE, Wallace M, Wang C, Moeller D, Korpics J, Novosad SA, Benowitz I, Jacobs MW, Dasari VS, Patel MT, Kauerauf J, Charles EM, Ezike NO, Chu V, Midgley CM, Rolfes MA, Gerber SI, Lu X, Lindstrom S, Verani JR, Layden JE, Illinois COVID-19 Investigation Team (2020). First known person-to-person transmission of severe acute respiratory syndrome coronavirus 2 (SARS-CoV-2) in the USA. Lancet.

[9] Xu Z, Tang Y, Huang Q, Fu S, Li X, Lin B (2021). Systematic review and subgroup analysis of the incidence of acute kidney injury (AKI) in patients with COVID-19. BMC Nephrol.

[10] Chen YT, Shao SC, Hsu CK, Wu IW, Hung MJ, Chen YC (2020). Incidence of acute kidney injury in COVID-19 infection: a systematic review and meta-analysis. Crit Care.

[11] Nasiri MJ, Haddadi S, Tahvildari A, Farsi Y, Arbabi M, Hasanzadeh S (2020). COVID-19 Clinical Characteristics, and Sex-Specific Risk of Mortality: Systematic Review and Meta-Analysis. Front Med (Lausanne).

[12] Zhang Z, Zhang L, Zha D, Hu C, Wu X (2020). Clinical characteristics and risks of Chinàs 2019 novel coronavirus patients with AKI: a systematic review and meta-analysis. Ren Fail.

[13] Fu EL, Janse RJ, de Jong Y, van der Endt VH, Milders J, van der Willik EM (2020). Acute kidney injury and kidney replacement therapy in COVID-19: a systematic review and meta-analysis. Clin Kidney J.

[14] Brasil. Ministério da Saúde (2020). COVID-19 no Brasil.

[15] Sun P, Qie S, Liu Z, Ren J, Li K, Xi J (2020). Clinical characteristics of hospitalized patients with SARS-CoV-2 infection: a single arm meta-analysis. J Med Virol.

[16] Wang D, Hu B, Hu C, Zhu F, Liu X, Zhang J (2020). Clinical Characteristics of 138 Hospitalized Patients With 2019 Novel Coronavirus-Infected Pneumonia in Wuhan, China. JAMA.

[17] Wu C, Chen X, Cai Y, Xia J, Zhou X, Xu S (2020). Risk Factors Associated With Acute Respiratory Distress Syndrome and Death in Patients With Coronavirus Disease 2019 Pneumonia in Wuhan, China. JAMA Intern Med.

[18] Cheng Y, Luo R, Wang K, Zhang M, Wang Z, Dong L (2020). Kidney disease is associated with in-hospital death of patients with COVID-19. Kidney Int.

[19] Zhou F, Yu T, Du R, Fan G, Liu Y, Liu Z (2020). Clinical course and risk factors for mortality of adult inpatients with COVID-19 in Wuhan, China: a retrospective cohort study. Lancet.

[20] Chen N, Zhou M, Dong X, Qu J, Gong F, Han Y (2020). Epidemiological and clinical characteristics of 99 cases of 2019 novel coronavirus pneumonia in Wuhan, China: a descriptive study. Lancet.

[21] Huang C, Wang Y, Li X, Ren L, Zhao J, Hu Y (2020). Clinical features of patients infected with 2019 novel coronavirus in Wuhan, China. Lancet.

[22] Huang D, Lian X, Song F, Ma H, Lian Z, Liang Y (2020). Clinical features of severe patients infected with 2019 novel coronavirus: a systematic review and meta-analysis. Ann Transl Med.

[23] Arentz M, Yim E, Klaff L, Lokhandwala S, Riedo FX, Chong M (2020). Characteristics and Outcomes of 21 Critically Ill Patients With COVID-19 in Washington State. JAMA.

[24] Cummings MJ, Baldwin MR, Abrams D, Jacobson SD, Meyer BJ, Balough EM (2020). Epidemiology, clinical course, and outcomes of critically ill adults with COVID-19 in New York City: a prospective cohort study. Lancet.

[25] Hirsch JS, Ng JH, Ross DW, Sharma P, Shah HH, Barnett RL, Hazzan AD, Fishbane S, Jhaveri KD, Northwell COVID-19 Research Consortium; Northwell Nephrology COVID-19 Research Consortium (2020). Acute kidney injury in patients hospitalized with COVID-19. Kidney Int.

[26] Pan XW, Xu D, Zhang H, Zhou W, Wang LH, Cui XG (2020). Identification of a potential mechanism of acute kidney injury during the COVID-19 outbreak: a study based on single-cell transcriptome analysis. Intensive Care Med.

[27] Perico L, Benigni A, Remuzzi G (2020). Should COVID-19 Concern Nephrologists? Why and to what extent? The emerging impasse of angiotensin blockade. Nephron.

[28] Ponce D (2020). The impact of coronavirus in Brazil: politics and the pandemic. Nat Rev Nephrol.

[29] Mehta RL, Kellum JA, Shah SV, Molitoris BA, Ronco C, Warnock DG, Levin A, Acute Kidney Injury Network (2007). Acute Kidney Injury Network: report of an initiative to improve outcomes in acute kidney injury. Crit Care.

[30] Chang CH, Fan PC, Chang MY, Tian YC, Hung CC, Fang JT (2014). Acute kidney injury enhances outcome prediction ability of sequential organ failure assessment score in critically ill patients. PLoS One.

[31] Caires RA, Abdulkader RC, Costa E Silva VT, Ferreira GS, Burdmann EA, Yu L (2016). Sustained low-efficiency extended dialysis (SLED) with single-pass batch system in critically-ill patients with acute kidney injury (AKI). J Nephrol.

[32] Himmelfarb J, Ikizler TA (2007). Acute kidney injury: changing lexicography, definitions, and epidemiology. Kidney Int.

[33] Bellomo R, Ronco C, Mehta RL, Asfar P, Boisramé-Helms J, Darmon M (2017). Acute kidney injury in the ICU: from injury to recovery: reports from the 5th Paris International Conference. Ann Intensive Care.

[34] Bellomo R, Kellum JA, Ronco C, Wald R, Martensson J, Maiden M (2017). Acute kidney injury in sepsis. Intensive Care Med.

[35] Bucuvic EM, Ponce D, Balbi AL (2011). Fatores de risco para mortalidade na lesão renal aguda. Rev Assoc Med Bras.

[36] Ponce D, Zamoner W, Batistoco MM, Balbi A (2020). Changing epidemiology and outcomes of acute kidney injury in Brazilian patients: a retrospective study from a teaching hospital. Int Urol Nephrol.

[37] Sabaghian T, Kharazmi AB, Ansari A, Omidi F, Kazemi SN, Hajikhani B (2022). COVID-19 and Acute Kidney Injury: a Systematic Review. Front Med (Lausanne).

[38] Nadim MK, Forni LG, Mehta RL, Connor MJ, Liu KD, Ostermann M (2020). COVID-19-associated acute kidney injury: consensus report of the 25th Acute Disease Quality Initiative (ADQI) Workgroup. Nat Rev Nephrol.

[39] Pei G, Zhang Z, Peng J, Liu L, Zhang C, Yu C (2020). Renal Involvement and Early Prognosis in Patients with COVID-19 Pneumonia. J Am Soc Nephrol.

[40] Mahalingasivam V, Su G, Iwagami M, Davids MR, Wetmore JB, Nitsch D (2022). COVID-19 and kidney disease: insights from epidemiology to inform clinical practice. Nat Rev Nephrol.

[41] Mohamed MM, Lukitsch I, Torres-Ortiz AE, Walker JB, Varghese V, Hernandez-Arroyo CF (2020). Acute Kidney Injury Associated with Coronavirus Disease 2019 in Urban New Orleans. Kidney360.

[42] Husain-Syed F, Wilhelm J, Kassoumeh S, Birk HW, Herold S, Vadász I (2020). Acute kidney injury and urinary biomarkers in hospitalized patients with coronavirus disease-2019. Nephrol Dial Transplant.

[43] Lowe R, Ferrari M, Nasim-Mohi M, Jackson A, Beecham R, Veighey K, Cusack R, Richardson D, Grocott M, Levett D, Dushianthan A, University Hospital Southampton Critical Care Team and the REACT COVID investigators (2021). Clinical characteristics and outcome of critically ill COVID-19 patients with acute kidney injury: a single centre cohort study. BMC Nephrol.

[44] Menez S, Parikh CR (2021). Overview of acute kidney manifestations and management of patients with COVID-19. Am J Physiol Renal Physiol.

[45] Zamoner W, Santos CA, Magalhães LE, de Oliveira PG, Balbi AL, Ponce D (2021). Acute Kidney Injury in COVID-19: 90 Days of the Pandemic in a Brazilian Public Hospital. Front Med (Lausanne).

[46] Magalhães LE, de Oliveira PG, Favarin AJ, Yuasa BK, Cardoso PA, Zamoner W, Oliveira PGSd, Favarin AJ, Zamoner W, Santos CAdS, Andriolo P, Yuasa KB, Zamoner W, Ponce D (2022). Acute kidney injury in coronavirus infectious disease: a study of incidence, risk factors, and prognosis during the first wave of the disease in Brazil. Int Urol Nephrol.

[47] Schaubroeck H, Vandenberghe W, Boer W, Boonen E, Dewulf B, Bourgeois C (2022). Acute kidney injury in critical COVID-19: a multicenter cohort analysis in seven large hospitals in Belgium. Crit Care.

[48] Hoste EA, Bagshaw SM, Bellomo R, Cely CM, Colman R, Cruz DN (2015). Epidemiology of acute kidney injury in critically ill patients: the multinational AKI-EPI study. Intensive Care Med.

[49] Kudose S, Batal I, Santoriello D, Xu K, Barasch J, Peleg Y (2020). Achados de biópsia renal em pacientes com COVID-19. J Am Soc Nephrol.

[50] Santoriello D, Khairallah P, Bomback AS, Xu K, Kudose S, Batal I (2020). Achados de patologia renal pós-morte em pacientes com COVID-19. J Am Soc Nephrol.

[51] Barretti P, Soares VA (1997). Acute renal failure: clinical outcome and causes of death. Ren Fail.

[52] Pombo CM, Almeida PC, Rodrigues JL (2010). Conhecimento dos profissionais de saúde na Unidade de Terapia Intensiva sobre prevenção de pneumonia associada à ventilação mecânica. Cien Saude Colet.

[53] Macedo E, Mehta RL (2011). When should renal replacement therapy be initiated for acute kidney injury?. Semin Dial.

[54] Ostermann M, Joannidis M, Pani A, Floris M, De Rosa S, Kellum JA, Ronco C, 17th Acute Disease Quality Initiative (ADQI) Consensus Group (2016). Patient Selection and Timing of Continuous Renal Replacement Therapy. Blood Purif.

[55] Annigeri RA, Ostermann M, Tolwani A, Vazquez-Rangel A, Ponce D, Bagga A (2017). Renal support for acute kidney injury in the developing world. Kidney Int Rep.

